# Investigation of an outbreak of disease compatible with scurvy in a male penitentiary in the state of Ceará, Brazil, 2019-2020: a case-control study

**DOI:** 10.1590/S2237-96222023000200020

**Published:** 2023-09-18

**Authors:** Leonardo José Alves de Freitas, Fernanda Sindeaux Camelo, Maria Júlia Araújo Borges, Ricristhi Gonçalves de Aguiar Gomes, Magda Moura de Almeida, Danniely Carolinne Soares da Silva, Márcio Henrique de Oliveira Garcia, Elizabeth David dos Santos

**Affiliations:** 1 Ministério da Saúde, Programa de Treinamento em Epidemiologia Aplicada aos Serviços do Sistema Único de Saúde, Brasília, DF, Brazil; 2 Secretaria da Saúde do Ceará, Secretaria Executiva de Vigilância em Saúde, Fortaleza, CE, Brazil; 3 Universidade Federal do Ceará, Departamento de Saúde Comunitária, Fortaleza, CE, Brazil; 4 Ministério da Saúde, Coordenação-Geral de Vigilância de Zoonoses e Doenças de Transmissão Vetorial, Brasília, DF, Brazil

**Keywords:** Scurvy, Hypovitaminosis, Prisons, Field epidemiology, Disease outbreaks, Case-control studies, Escorbuto, Hipovitaminosis, Prisiones, Epidemiología de campo, Brotes de enfermedades, Estudios de casos y controles, Escorbuto, Hipovitaminose, Penitenciária, Epidemiologia de Campo, Surtos de Doenças, Estudos de Casos e Controles

## Abstract

**Objective:**

to identify the occurrence of an outbreak compatible with scurvy and exposure factors associated with typical signs/symptoms of hypovitaminosis that occurred in a male penitentiary in Ceará, Brazil between 2019-2020.

**Methods:**

this was a population-based case-control study; we used clinical records and interviews with compatible cases - based on sign/symptom onset during the study period - and with controls; we carried out multivariate analysis.

**Results:**

out of 62 cases, mean age was 40.6 years (SD = 10.8); main signs/symptoms were edema and pain in the lower limbs (100.0%), difficulty in walking (91.9%), hematoma/ecchymosis in the lower limbs (90.3%) and fever (88.7%); we identified being over 40 years old as an associated factor (aOR = 1.10; 95%CI 1.05;1.17; p-value = 0.001); and as protective factors: working (aOR = 0.11; 95%CI 0.03;0.36; p-value < 0.001) and taking part in classes (aOR = 0.21; 95%CI 0.08;0.59; p-value = 0.003) in the prison.

**Conclusion:**

we considered the penitentiary outbreak to be compatible with scurvy due to characteristic signs/symptoms, associated with the identified factors; we recommended regular provision of a diet rich in vitamin C to all male inmates and clinical follow-up of cases.

## Study contributions

**Main results:**

An outbreak of illness compatible with scurvy occurred among male prison inmates; most frequent signs/symptoms were edema and pain in lower limbs, difficulty in walking and hematoma/ecchymosis; the associated factor was age > 40 years.

**Implications for services:**

The results can contribute to the identification and description of scurvy outbreaks in other contexts and territories. Epidemiological studies of events like this enhance the practice of field epidemiology in health services.

**Perspectives:**

It is expected that the prevention of hypovitaminosis, such as scurvy, will be based on public policies aimed at the population deprived of liberty; and that the capabilities of health services to detect and respond to cases of the disease will be improved.

## RESUMEN

**Objetivo:**

identificar la ocurrencia del brote compatible con escorbuto y los factores de exposición asociados con signos/síntomas típicos de hipovitaminosis que ocurrieron en una penitenciaría masculina en Ceará, Brasil entre 2019-2020.

**Métodos:**

estudio poblacional de casos y controles; se utilizaron historias clínicas y entrevistas con casos compatibles, con inicio de signos/síntomas durante el período de estudio, y controles; se realizó análisis multivariable.

**Resultados:**

de los 62 casos, edad media fue 40,6 años (DE = 10,8); principales signos/síntomas fueron edema y dolor en miembros inferiores (100,0%), dificultad para caminar (91,9%), hematoma/equimosis en miembros inferiores (90,3%) y fiebre (88,7%); la edad mayor de 40 años se identificó como factor asociado (ORa = 1,10; IC_95%_ 1,05;1,17; p-valor = 0,001); y como factores protectores: trabajo (ORa = 0,11; IC_95%_ 0,03;0,36; p-valor < 0,001) y participación en clases (ORa = 0,21; IC_95%_ 0,08;0,59; p-valor = 0,003) dentro del centro penitenciario.

**Conclusión:**

el brote en centro penitenciario fue considerado compatible con escorbuto debido a los signos/síntomas característicos, asociados a los factores identificados; se recomendó oferta regular de dieta rica en vitamina C a todos los internos y seguimiento clínico de los casos.

## RESUMO

**Objetivo:**

identificar ocorrência de surto compatível com escorbuto e fatores de exposição associados aos sinais/sintomas típicos de hipovitaminose, em penitenciária masculina, Ceará, Brasil, 2019-2020.

**Métodos:**

estudo de caso-controle populacional; foram utilizados registros clínicos e entrevistas com casos compatíveis - sinais/sintomas iniciados no período - e com controles; realizou-se análise multivariável.

**Resultados:**

62 casos; idade média de 40,6 anos (DP = 10,8); principais sinais/sintomas foram edema e dor em membros inferiores (100,0%), dificuldade para deambular (91,9%), hematoma/equimose em membros inferiores (90,3%), febre (88,7%); identificou-se, como fator de exposição, média de idade > 40 anos (ORa = 1,10; IC_95%_ 1,05;1,17; p-valor = 0,001); e como fatores protetores, trabalho (ORa = 0,11; IC_95%_ 0,03;0,36; p-valor < 0,001) e participação em aulas (ORa = 0,21; IC_95%_ 0,08;0,59; p-valor = 0,003) dentro da penitenciária.

**Conclusão:**

surto da penitenciária compatível com escorbuto pelos sinais/sintomas característicos, associados aos fatores identificados; recomendou-se oferta regular de dieta rica em vitamina C para todos os internos e acompanhamento clínico dos casos.

## INTRODUCTION

Scurvy is a form of hypovitaminosis, characterized by ascorbic acid (vitamin C) deficiency. It develops in a period of between one and three months, among individuals or communities whose diet is devoid of fresh vegetables and fruit.^1^

The disease progresses with presence of clinical manifestations, such as lassitude, anorexia, dizziness, fatigue, hemorrhagic signs - including bleeding gums, petechiae and ecchymosis -, edema, difficulty in healing, skin changes and infections, which can lead to death.^2^

Scurvy is currently considered a rare disease, although it is still identified in infants, elderly people living alone, the indigent, alcoholics and people with very limited diets.^1^ ^),(^ ^3^ ^)^ Outbreaks of scurvy can still occur, especially among vulnerable people such as refugees and populations deprived of freedom. In 2016, an outbreak in an Ethiopian prison was investigated, where the occurrence of cases and deaths related to the disease was described.^3^

The description of the occurrence of nutritional diseases in people deprived of liberty demonstrates the importance of micronutrients in human health.^4^ Knowledge about the epidemiological profile of cases and identification of factors that favor the occurrence of scurvy outbreaks in the Brazilian scenario, as well as reporting on related technical information, contributes to the formulation of effective actions for scurvy prevention and control. 

This study aimed to identify the occurrence of an outbreak of disease compatible with scurvy and exposure factors associated with the typical signs and symptoms of hypovitaminosis, which occurred in a male penitentiary in the state of Ceará, Brazil, between 2019 and 2020.

## METHODS

*Design*

This was an unpaired population-based case-control study, that included the period between October 1, 2019 and March 20, 2020. The field epidemiology investigation took place from March 8 to 20, 2020. 

*Background*

In February 2020, the Ceará State Health Department (SESA/CE) Center for Strategic Information on Health Surveillance was notified about the occurrence of an unknown disease, observed among people deprived of liberty, inmates of a male state penitentiary, who had, in particular, clinical manifestations such as edema, pain and hematoma/ecchymosis in their lower limbs. 

In March 2020, support was requested from the Health and Environmental Surveillance Secretariat of the Brazilian Ministry of Health (SVSA/MS), through its Brazil Field Epidemiology Training Program (Brazil FETP, “EpiSUS-Avançado”), for epidemiological investigation of the event. By that time, 49 suspected cases had been reported.

The first diagnostic hypotheses raised by the attending physicians were: accident caused by a venomous animal; deep vein thrombosis; exogenous intoxication; trauma; filariasis; leptospirosis; arboviruses; and spotted fever. Based on discussions between the teams involved, the attending physicians began to define, as a diagnostic hypothesis, vitamin deficiency, especially vitamin C.

Following the hypothesis of hypovitaminosis, the teams from the reference hospitals in Fortaleza and the prison Health Unit began supplementing micronutrients - vitamins C, B, K, folic acid, ferrous sulfate - and other symptomatic treatments among the cases.

The penitentiary consisted of nine wings, each with 19 cells measuring about six square meters. The company responsible for providing food at the prison was outsourced and was hired by the Ceará state government in June 2019 under a one-year contract. 

*Participants*

The inmates of the male prison were included in the study.^5^ The official capacity of the prison unit was 1,016 places; however, it housed 2,179 inmates, according to a census carried out by the prison police on March 19, 2020.^6^ ^),(^ ^7^ The counting of inmates was a daily procedure at the prison. 

The definitions adopted for this investigation were: 

a) suspected case - inmate with a record of clinical care in the prison because, in the period from October 1, 2019 to March 20, 2020, he had had at least one of the following signs/symptoms: (i) edema in lower limbs, (ii) hematoma/ecchymosis in lower limbs, (iii) pain in lower limbs, (iv) insomnia, (v) irritation/agitation, (vi) hyporexia/lack of appetite, (vii) gingival alterations (hypertrophy, bleeding), (viii) asthenia/fatigue/tiredness/adynamia, (ix) difficulty in walking, (x) heart failure, (xi) signs of inflammation (pain, redness, heat) in lower limbs, (xii) stiffness in lower limbs or (xiii) difficulty breathing; 

b) case compatible with scurvy - suspected case, with edema in lower limbs and at least one of the following signs/symptoms: (i) pain in lower limbs, (ii) hematoma/ecchymosis in lower limbs, (iii) signs of inflammation (pain, redness, heat) in lower limbs, (iv) stiffness in lower limbs, (v) difficulty in walking, (vi) bleeding gums or fever; and/or (vii) improved clinical picture (positive therapeutic response) after receiving ascorbic acid supplementation; and 

c) control - prison inmate who, in the period from October 1, 2019 to March 20, 2020, did not show the signs and symptoms reported by cases compatible with scurvy. 

The case/control ratio was 1:1.2 - 62 compatible cases/72 controls -, based on the number of cases identified and the safety conditions for selecting controls. 

The selection of controls was carried out in two phases. In the first phase, based on a list of all inmates who met the definition of control, systematic selection was performed, including all nine wings of the prison: approximately eight controls were selected from each of the wings, from a list of inmates in the order identified by the prison census. The second selection phase was carried out by convenience: the prison police themselves, in charge of cell security, selected one inmate per alternating cell from each wing.

Inmates who had other clinical diagnoses, those who showed signs of disorientation when answering the questions, and those in isolation were excluded from the study. Controls who had been deprived of liberty for less than two months in the prison (July to December 2019) were also excluded. 

*Variables*

The time and place variables analyzed were:

- approximate date of sign and symptom identification; 

- total time of deprivation of liberty [mean time (in years) the inmate had been deprived of liberty (including in other prison units) until the date of the interview]; 

- time of deprivation of liberty in the prison [mean time (in years) the inmate had been deprived of liberty at that prison until the date of the interview]; and

- number of inmates per cell.

The sociodemographic variables were: 

- race/skin color (White; mixed race; Black);

- schooling (illiterate; elementary education; high school education; higher education); 

- age (last birthday); and

- age group (in years: 20-29; 30-39; 40-49; 50-59; and 60-65).

The clinical variables included were: 

- signs and symptoms presented;

- comorbidities (yes or no; type);

- disability (physical, hearing or vision);

- long-term medication (yes or no; type);

- hospitalization (yes or no);

- medication and vitamins received (yes or no; type);

- progression following medication or vitamin supplementation; and

- results of laboratory tests (human immunodeficiency virus: HIV); viral hepatitis; filariosis; imaging examination; and vitamin B and C levels).

The variables regarding life habits before being deprived of liberty were: 

- tobacco smoking;

- alcoholism; and

- use of other substances. 

The variables regarding inmates’ routines and daily living conditions in the prison during the study period were: 

- receives visits (yes or no);

- degree of kinship of visitors;

- frequency of visits (in days);

- external source of food (yes or no); 

- number of meals offered by the prison administration per day;

- kinds of food served as meals;

- taking part in classes in the prison (yes or no);

- working in the prison (yes or no);

- working serving meals (yes or no); and

- undertaking physical exertion (yes or no).

*Data sources and measurement*

The primary data were collected during face-to-face interviews, by means of a semi-structured questionnaire. The interviews were conducted by SESA/CE health professionals and the SVSA/MS team, with the support of public security and prison staff. The “external source of food” variable referred to the types of food or drinks received by the inmates during visits, every 15-20 days.

Secondary data were obtained through a retrospective and active search for cases, based on the review of electronic medical records of those with written records of attendance at the prison clinic and at Ceará prison complex health services (outpatient or inpatient). Medical records and inmates care records at the reference hospitals in Fortaleza (Hospital São José de Doenças Infecciosas and Hospital Geral de Fortaleza) were also used as data sources. Subsequently, a list of cases was created for the purpose of epidemiological investigation.

According to the diagnostic hypotheses of the health care teams at the Hospital São José de Doenças Infecciosas and the Hospital Geral de Fortaleza, the main laboratory tests requested for the inmates treated were for HIV, viral hepatitis, filariasis, imaging examinations and vitamin B and C levels.

*Bias control*

In order to reduce information and memory bias, interviewers were trained with the aim of standardizing data collection and recording. Printed calendars were also used to help the inmates remember dates.

*Sample size*

All case-inmates who had the signs and symptoms specified in the compatible case definition were included, based on identification by retrospective and active searching. The number of controls was achieved according to control selection and the security conditions available during the epidemiological investigation.

*Statistical methods*

We used descriptive statistics, by means of absolute and relative frequencies, as well as central tendency and dispersion measurements. Analytical statistics were used in the bivariate analysis, by means of Pearson’s chi-square test and Fisher’s exact test for categorical variables, as well as Student’s t-test for continuous variables. 

In order to reduce possible confounding factors, we performed multivariate analysis. Based on criteria of causality and natural history of the disease, exposure variables with a p-value ≤ 0.2 in the bivariate analysis were included in a logistic regression model, using a 95% confidence interval (95%CI). 

Association was measured using the crude odds ratio (OR) for bivariate analysis, and the adjusted odds ratio (aOR) for multivariate analysis. A p-value < 0.05 was used as the significance level in both forms of analysis. 

We used Epi Info 7.2.3.1 and Microsoft Excel 2016® to process and analyze the data.

*Ethical aspects*

The study was characterized as an epidemiological surveillance action, as provided for by Law No. 8080, dated September 19, 1990. Verbal consent was required for participation in the interviews, and participants were informed about the objectives of the epidemiological investigation, as well as the purpose and confidentiality of data collection. The study project was approved by the National Health Council Research Ethics Committee on October 3, 2021, as per Certificate of Submission for Ethical Appraisal No. 49187421.0.0000.0008 and Opinion No. 5.013.046.

## RESULTS

**Figure 1:**
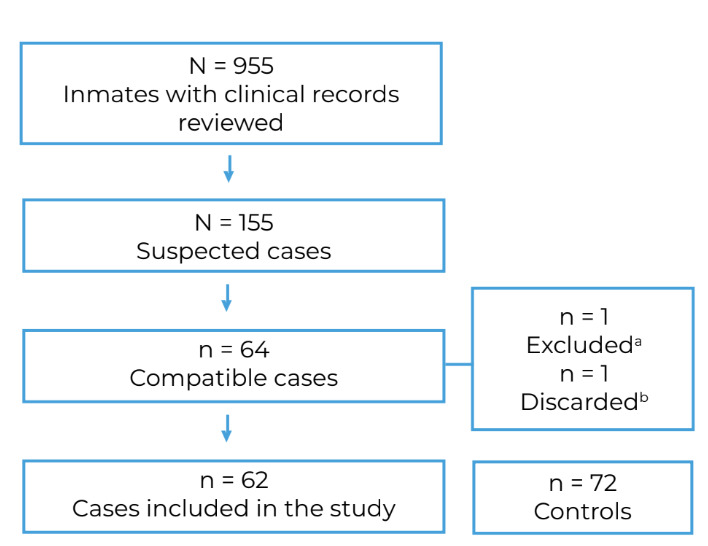
Flow of retrospective and active search for cases compatible with scurvy and controls among inmates of a male penitentiary, Ceará, Brazil, 2019-2020

In all, the clinical records of 995 inmates were reviewed and 155 of them met the definition of a suspected case of scurvy. After reviewing clinical records and medical records, 64 inmates met the compatible case definition. Sixty-four interviews were conducted, leading to one case being excluded and another being discarded. In the end, 62 compatible cases and 72 controls were included in the study (Figure 1).

a) Case excluded for being disorientated when answering the questions; b) Case discarded due to diagnosis of recurrent erysipelas.

**Figure 2:**
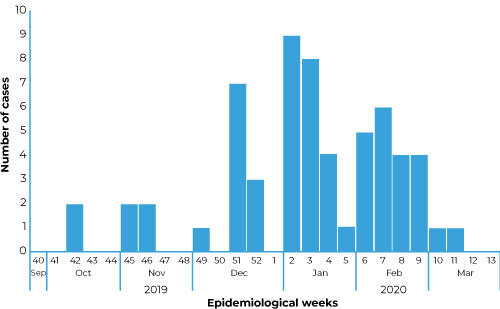
Distribution of cases compatible with scurvy (N = 60) by epidemiological week of sign and symptom identification among inmates of a male penitentiary, Ceará, Brazil, 2019-2020

The first cases compatible with scurvy reported signs and symptoms around October 15, 2019 (Epidemiological Week 42: October 13 to 19, 2019) (Figure 2). The mean total time of deprivation of liberty of the cases was 4.0 years [standard deviation (SD) = ±4.1]; and the mean time of deprivation of liberty in the prison in question was 1.6 years (SD = ±1.2).

The reported average number of inmates per cell was 21.3 (SD = ±8.6). From July to December 2019, inmates were reorganized between cells, within the same wings. 

Of the 62 compatible cases, 47 stated that they were of mixed race and 41 reported having elementary education. Their mean age was 40.6 years (SD = ±10.8), while the most affected age group ranged from 30 to 39 years (n = 22). Among the same compatible cases, 57 self-reported being of mixed race/skin color; and 52 as having completed elementary education . The mean age of the controls was 32.0 years (SD = ±7.1), with the predominant age group also being 30 to 39 years (Table 1). 

**Table 1:** Distribution of cases compatible with scurvy, by race/skin color, schooling, age group and signs and symptoms presented among inmates of a male penitentiary, Ceará, Brazil, 2019-2020

Characteristics	Cases n = 62	Controls n = 72
Race/skin color		
Mixed race	47	57
White	14	14
Black	1	1
Schooling		
Elementary education	41	52
Illiterate	17	6
High school education	4	12
Higher education	-	2
Age group (last birthday)		
20-29	9	30
30-39	22	31
40-49	16	11
50-59	12	-
60-65	3	-
Signs and symptoms		
Edema in lower limbs	62	-
Pain in lower limbs	62	-
Difficulty in walking	57	-
Hematoma/ecchymosis in lower limbs	56	-
Fever	55	-
Asthenia	54	-
Stiffness in lower limbs	51	-
Hyporexia	45	-
Signs of inflammation in lower limbs	42	-
Bleeding gums	30	-

**Figure 3:**
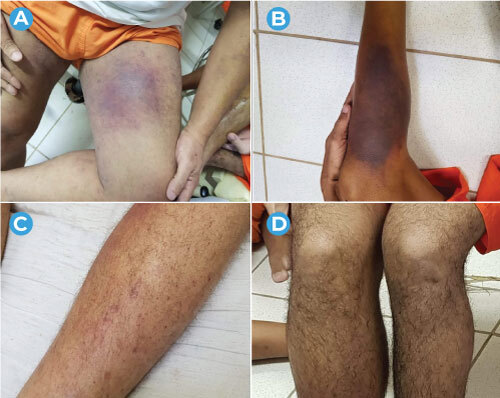
Clinical manifestations of cases compatible with scurvy, showing extensive hematomas/ecchymosis (A and B), petechiae (C) and edema (D) in the lower limbs, among inmates of a male penitentiary, Ceará, Brazil, 2019-2020

Source: Ceará Prison Administration Department and Ceará Health Department.

The most frequent signs and symptoms were edema and pain in the lower limbs, reported by all 62 cases; difficulty in walking, reported by 57; and hematoma/ecchymosis in the lower limbs, reported by 56 (Figure 3).

Compatible cases also had other signs and symptoms, such as: dizziness in 47, weight loss in 46, chills and headache in 41, cough in 38, diarrhea in 36, abdominal discomfort in 32, dyspnea in 31, constipation in 22, chest pain in 19 and edema in the upper limbs in 14 cases.

The most reported comorbidities were hypertension, in 13 cases; and tuberculosis and gastritis, in 7 cases. Of the total number of compatible cases, 22 reported having a disability: 10 reported visual impairment; 8, physical disability; and 4, hearing loss. Seventeen reported continuous use of some type of medication.

Of the 62 compatible cases, 41/57 were admitted to one of the referral hospitals. No deaths due to scurvy were identified.

Among the compatible cases, 60 reported having received medication due to scurvy and of these, 55 stated having received some type of vitamin and 58 reported improvement in their clinical picture after vitamin supplementation, mainly vitamin C. 

Still with regard to compatible cases, 38 reported having been smokers; and 55, consumers of alcoholic beverages; 36 said they used other substances before deprivation of liberty, among the most mentioned were marijuana reported by 27/36, crack reported by 13/36 and cocaine reported by 10/36.

Thirty reported that they received visits before becoming ill due to scurvy, 28 from family members and 2 from lawyers, every 15 to 20 days. Of those who received visits, 26/30 also referred to them as an external source of food, mainly: sandwiches (20/26); soda (19/26); apples (16/26); and water (12/26).

Thirty-seven stated that they consumed the food offered at meals every day in the prison. A total of 24 compatible cases responded that they did not usually eat all meals and of these, 14/24 reported that when the food reached them it had gone off or was spoiled.

Among the foods offered by the contracted company, rice, beans, pasta, farofa (fried cassava flour), feijoada (pork and bean stew), chicken thigh/drumstick and sausage were mentioned, followed by baião de dois (rice and beans), boned pork, stew/buchada (protein-rich food based on white viscera, such as beef tripe) and gizzard in sauce. The least mentioned foods were raw salad, meatballs/chopped meat, bone-in pork and bone-in beef; the latter two items had been excluded from the menu for security reasons. 

Provision of fruit juice was not reported by any of the cases, nor was fruit. Among cooked vegetables, the cases mentioned that potatoes, cassava, pumpkin and chayote were served. As for raw salad, carrots and beetroot were mentioned, despite the small amount of these foods.

Twelve microfilaria tests were requested: three had negative results. Four HIV tests and four viral hepatitis tests were performed, also with negative results. Twelve samples were collected for testing for vitamin C levels, but the tests were not performed due their unavailability in public laboratories. Some cases had other imaging examinations but the results did not indicate signs of deep vein thrombosis. 

The “age” variable (at last birthday) remained independently associated with the chance of scurvy occurring (aOR = 1.10; 95%CI = 1.05;1.17; p-value = 0.001). The variables that, after multivariate analysis, remained independently associated with a reduction in the chance of scurvy occurring were working (aOR = 0.11; 95%CI = 0.03;0.36; p-value < 0.001) and taking part in classes (aOR = 0.21; 95%CI = 0.08;0.59; p-value = 0.003) in the prison (Table 2).

**Table 2:** Bivariate and multivariate analyses of exposures possibly associated with the occurrence of cases compatible with scurvy among inmates (N = 134) of a male penitentiary, Ceará, Brazil, 2019-2020

Variable	n	Crude OR^a^ (95%CI^c^)	p-value^d^	Adjusted OR^b^ (95%CI^c^)	p-value^d^
Age (last birthday)					
> 40	61	4.41 (2.13;9.14)	< 0.001	1.10 (1.05;1.17)	0.001
≤ 40	73	1.00			
Working in the prison					
Yes	36	0.19 (0.08;0.47)	< 0.001	0.11 (0.03;0.36)	< 0.001
No	98	1.00			
Taking part in classes in the prison					
Yes	45	0.24 (0.11;0.54)	< 0.001	0.21 (0.08;0.59)	0.003
No	89	1.00			
External source of food					
Yes	69	0.49 (0.24;0.97)	0.039	0.49 (0.19;1.27)	0.143
No	65	1.00			
Alcoholism					
Yes	111	2.62 (0.95;7.19)	0.055	2.68 (0.72;9.99)	0.141
No	22	1.00			
Disability (physical, hearing or vision)					
Yes	34	2.20 (0.99;4.91)	0.050	2.38 (0.77;7.33)	0.130
No	97	1.00			
Schooling					
Elementary/high school/higher education	111	0.24 (0.09;0.66)	< 0.001	0.55 (0.12;2.52)	0.446
Illiterate	23	1.00			
Long-term medication					
Yes	28	2.20 (0.94;5.19)	0.065	1.31 (0.41;4.13)	0.650
No	102	1.00			
Time of deprivation of liberty in the prison (in years)					
> 2	72	2.73 (1.34;5.58)	0.005	0.85 (0.31;2.35)	0.756
≤ 2	59	1.00			
Working serving meals					
Yes	18	0.37 (0.12;1.11)	0.067	0.91 (0.07;11.74)	0.943
No	120	1.00			
Undertaking physical exertion					
Yes	46	0.56 (0.27;1.16)	0.118	0.92 (0.32;2.72)	0.892
No	88	1.00			

a) Crude OR: Crude odds ratio; b) adjusted OR: Adjusted odds ratio; c) 95%CI: 95% confidence interval; d) P-value via Pearson’s chi-square test, Fisher’s exact test and Student’s t-test.

## DISCUSSION

This study investigated one of the first outbreaks compatible with scurvy in Brazil, among the inmates of one of the largest male prisons in the state of Ceará, in the midst of the onset of the COVID-19 pandemic. This led to the suspension of the entry of people from outside into the prison, given the risk of transmission of the virus among inmates. 

Some possible limitations of this study should be mentioned: (i) memory bias, due to the difficulty of respondents remembering past dates and situations; (ii) information bias, due to the difficulty in obtaining information from inmates and lack of nutritional assessment of foods consumed before illness; and (iii) selection bias, due to the selection of controls by convenience by the prison police. 

Confirmation of the outbreak was based on clinical manifestations, dietary history and positive therapeutic response to treatment following administration of ascorbic acid.^8^ ^),(^ ^4^ ^)^ Laboratory measurement of vitamin C levels was not possible in this epidemiological investigation. However, in the case of scurvy in children, it has been found that vitamin C levels are variable, and that diagnosis requires more than this. Furthermore, clinical improvement of cases has been identified, with a rapid and complete reduction of signs and symptoms following vitamin C supplementation, this being a result that serves as an indication of this diagnostic-therapeutic procedure serving as the best evidence of scurvy.^9^

The most obvious signs and symptoms, such as pain, edema, hematoma/ecchymosis and petechiae in lower limbs, difficulty in walking and bleeding gums, were compatible with those also identified in an outbreak of scurvy in the general population of a region of Afghanistan, in 2002,^10^ and in people deprived of their liberty in Ethiopia, in 2021.^3^ Scurvy can manifest itself clinically, in a more nonspecific way, at the beginning of illness, including signs such as asthenia, fatigue, irritability, diarrhea and weight loss.^3^ ^),(^ ^10^ As such, the number of cases may have been underestimated, due to the difficulty inmates and health professionals had in detecting the first signs and symptoms. The possibilities of lack of other vitamins were not ruled out and therefore, given the situation found and the opportunities available, vitamin C, K and complex B supplementation was provided.

Contrary to the recommended daily intake of micronutrients, the most mentioned types of food indicated food monotony, with absence of fresh fruit, vegetables and fruit juice, which are important sources of vitamin C that could prevent cases of scurvy.^11^ ^),(^ ^12^

The main reason for not eating all the meals offered was because of food that had gone off or was spoiled, which may also have favored micronutrient deficiency, mainly vitamin C, leading to the occurrence of the outbreak.^13^

Recommended daily intake of vitamin C has been established at 75 mg/day for women and 90 mg/day for men. Just 10 mg/day can prevent scurvy symptoms, although this is not enough to maintain adequate reserves of vitamin C in the body. Recommended levels are usually achieved by consuming citrus fruits and fresh vegetables, such as tomatoes and leafy greens.^14^

As age increases, people tend to present subclinical diseases and mobility difficulties capable of influencing the concentration of ascorbic acid in the plasma.^15^ Institutionalized people, facing substandard diets, especially with regard to fruit and other fresh vegetables, are a risk group for developing scurvy and other nutritional deficiencies.^16^ ^),(^ ^17^ ^)^

Compatible cases were found in cells in all the prison wings, and were not concentrated in one particular place. In addition, inmates were transferred to other cells. When analyzing participation in classes and doing work inside the prison, the findings suggest that, somehow, some inmates were exposed to different and varied conditions of access to food and routines inside the cells and wings of the prison, in comparison to others, thus increasing or decreasing the likelihood of their becoming ill from scurvy.^15^ ^)-(^ ^17^

There were no associations between alcoholism and use of other substances before deprivation of liberty, long-term medication or disability (physical, hearing or vision). Health conditions such as these are considered risk factors for the occurrence of scurvy. As no association was found, this suggests that other forms of exposure may be involved in the prison environment.^10^ ^),(^ ^15^

The following recommendations were made to the prison and to the Ceará State Health Department: (i) regular provision of foods rich in vitamin C to all inmates; (ii) provision of medical and physiotherapy follow-up for scurvy cases; (iii) intensification of health surveillance actions, in order to identify health events early and adopt timely prevention and control measures; and (iv) carrying out sanitary inspection actions to ensure the quality of meals offered to inmates.

The epidemiological investigation provided a description of the compatible outbreak of scurvy, leading to the identification of conditions of vulnerability in the population deprived of liberty that influenced their illness. At the same time, the importance of vitamin supplementation, provided to all cases and other inmates, stands out, as do improvements in diet, which probably contributed to controlling the outbreak and preventing new cases after the collective and administrative health interventions conducted in the prison.

We conclude that the disease outbreak with signs and symptoms typical of hypovitaminosis that occurred in the prison was identified as being an outbreak of scurvy, given the characteristic signs and symptoms presented. The data revealed that being over 40 years old was a factor that favored exposure, while protective factors were working and attending classes in the prison. Although a relationship between the reversal of signs and symptoms after micronutrient supplementation, notably vitamin C, was not directly investigated, due to the epidemiological situation, increased daily provision of food sources containing micronutrients, such as fresh fruit and vegetables, was recommended regarding meals served to the inmates of this male penitentiary and other prisons in the state of Ceará, in Brazil.
